# Bacterial diversity in the honey sac during bee foraging on winter-flowering flora and dominant *Bacillus subtilis* inhibits *Hafnia alvei*

**DOI:** 10.3389/finsc.2025.1555434

**Published:** 2025-03-11

**Authors:** Miao Wang, Wenzheng Zhao, Danyin Zhou, Jian Huang

**Affiliations:** ^1^ College of Food Science and Technology, Yunnan Agricultural University, Kunming, China; ^2^ Faculty of Animal Science and Technology, Yunnan Agricultural University, Kunming, China

**Keywords:** honey sac, winter flowering flora, *Apis cerana*, *Apis mellifera*, *Bacillus subtilis*, bacteriostasis

## Abstract

**Background:**

The bacterial diversity of two bee species in the process of honey collection during the flowering season of three different floral sources in the winter was studied. The common bacterium in all samples was *Bacillus subtilis*.

**Methods:**

In the present study, we collected nectar, honey sacs, and fresh honey during the winter flowering season of *Agastache rugosa*, *Prunus cerasoides*, and *Brassica rapa*. The pure culture method was used to count and analyze the number of bacteria, they were identified using 16S rRNA sequencing, similarities were compared in NCBI, and the common dominant bacterial species *B. subtilis* in all samples using phylogenetic analysis and intersection analysis were determined to conduct further bacteriostatic experiments.

**Results:**

The results showed that the most abundant quantity of bacteria could be found in the honey sacs, compared to in nectar or fresh honey. At the same time, the highest abundance of bacteria could be found in the honey sacs of *A. cerana* when collected on *Brassica rapa*, while the highest abundance of bacteria could be found in the honey sacs of *A. mellifera* when collected on *Prunus cerasoides* and *Agastache rugosa*. A total of 33 bacterial species were isolated, with variations in their distribution across different sample types and sources. The inhibitory effect of 10^-1^-10^-5^ on *Hafnia alvei* by *B. subtilis* was very significant.

**Conclusions:**

*B. subtilis* was identified in all sample sources, indicating the potential importance of *B. subtilis* as a probiotic in the bee gut for honey production, and *B. subtilis* could promote the disease resistance and health of honeybees in winter.

## 1 Introduction

The gut microbiota of the honeybee has emerged as a key factor influencing the health and vitality of these essential pollinators (1). The symbiotic relationship between bees and their microbial passengers is integral to their ability to digest complex floral nectars and pollen, defend against pathogens, and adapt to environmental stressors (1–3). The composition of the bee gut microbiota is shaped by a variety of factors, including genetic, immunological, social status, and environmental interactions, which ultimately affect the bees’ capacity to perform their ecological roles effectively (4–6).

Among the environmental factors, the choice of floral resources is particularly influential. The nutritional and chemical properties of nectar and pollen can significantly affect the development and stability of the gut microbiota (7, 8). The foraging behavior of bees during different seasons, and their interaction with available flora, introduce temporal dimensions to this microbiota-environment relationship (9, 10).

Seasonal changes in floral availability prompt bees to forage on a variety of plant species, including those that flower in winter. However, only in a few places do researchers have the chance to study the bees foraging on flowers (11). These winter-flowering plants present unique challenges and opportunities for bees, given their distinct biochemical compositions and the bees’ need to adapt to colder temperatures and altered environmental conditions (9). The study of how bees and their microbiota respond to winter nectar sources is therefore critical to understanding the resilience of bee populations in the face of seasonal adversity.

Honey sacs act as a nectar sink and transmitter between floral nectar and freshly downloaded honey, and play a crucial role in filtering the flow of harmful substances to mid-gut or hind-gut with a special valve. The nectar is passed from the dancer bees, who first store the nectar, normally 10-50 uL, in their honey sac, to the next indoor mates during waggle dances (12). The indoor mates, based on the information from the passing nectar and dance, either save the nectar into a honey cell or are triggered by the dancer to fly out for more foraging. The honey sac, as the anterior part of a bee’s digestive system, introduces a microbial flora from the nectar during the process of nectar intake. Additionally, air is also mixed in during this process, so the honey sac is not a strictly anaerobic environment. As the nectar is regurgitated back into the honeycomb, both the nectar and the microorganisms from the honey sac are introduced into the cells. The honey sac, being a relatively open space, can also be colonized by aerobic bacteria such as *Bacillus subtilis*, which can inhibit pathogenic microorganisms at the front end of the digestive tract (13).

The present study focuses on the honeybee species *Apis cerana* and *A. mellifera*, which are known to forage on winter-flowering plants such as *Agastache rugosa*, *Prunus cerasoides*, and *Brassica rapa* (14, 15). These plants provide a vital food source during a period when other floral resources are scarce, especially in Yunnan, China. Unlike previous studies that focused on the diverse microbiome of raw honey, this study focused on diverse sources since this diversity may originate from pollen, nectar, air, the honeybee digestive tract (from mouth parts to the honey sac to the midgut and hindgut), contamination during processing by bees in the hive from fresh honey to ripe honey, and honey extraction by humans (16, 17). The research aims to elucidate the bacterial diversity within the nectar, honey sacs, and fresh honey collected by these bees from the above winter-flowering sources with less contamination from honey processing in the hive and from honey extraction by humans. By doing so, the study seeks to identify the key microbial constituents and their potential implications for bee health and honey quality, with a special focus on the microbiota effect on bee health and digestive effects (18).

*Hafnia alvei* is one of several *Enterobacteriaceae* species that are sporadically found in the gut of bees and may represent opportunistic pathogens (19). Indeed, good bee health is clearly linked to the stable functioning of ecosystems and indirectly to human well-being (20). *H. alvei* is a serious infectious disease that threatens bee populations, but it is mostly reported in *A. mellifera*. It has the characteristics of rapid spread, severe damage, long disease duration, and contamination of bee products (21). *H. alvei* is one of the pathogens causing septicemia in adult bees, and research reports that two bacterial members of the bee gut, *Gilliamella* and *Lactobacillus*, can clear *H. alvei* during invasion (22). In addition to isolating common dominant bacteria, this experiment also used *B. subtilis* to perform bacteriostatic experiments on hive honeydew, further demonstrating the defensive effect of *B. subtilis* in the honey sac against pathogenic bacteria.

Previous research has shed light on the general composition of bee gut microbiota and its role in honeybee biology. However, there is a dearth of knowledge regarding the specific dynamics of microbial communities as they progress from nectar through the honey production process, particularly during winter months. This study addresses this gap by examining the bacterial diversity at various stages of honey processing, offering a nuanced perspective on the interplay between bees, their microbiota, and the winter floral resources they exploit. Based on the current understanding of bee microbiota and the unique challenges posed by winter foraging, we learned much about the diversity of cultivable bacteria in honeybees during winter foraging, reflecting adaptation to the specific nutritional and chemical properties of winter nectar sources. We are also gaining a better understanding of the resistance of dominant bacteria to pathogens and the promotion of bee health, which may have implications for colony management and improving honey production during the winter season.

## 2 Materials and methods

### 2.1 Sample collection

#### 2.1.1 Nectar sample collection

**Figure 1:**
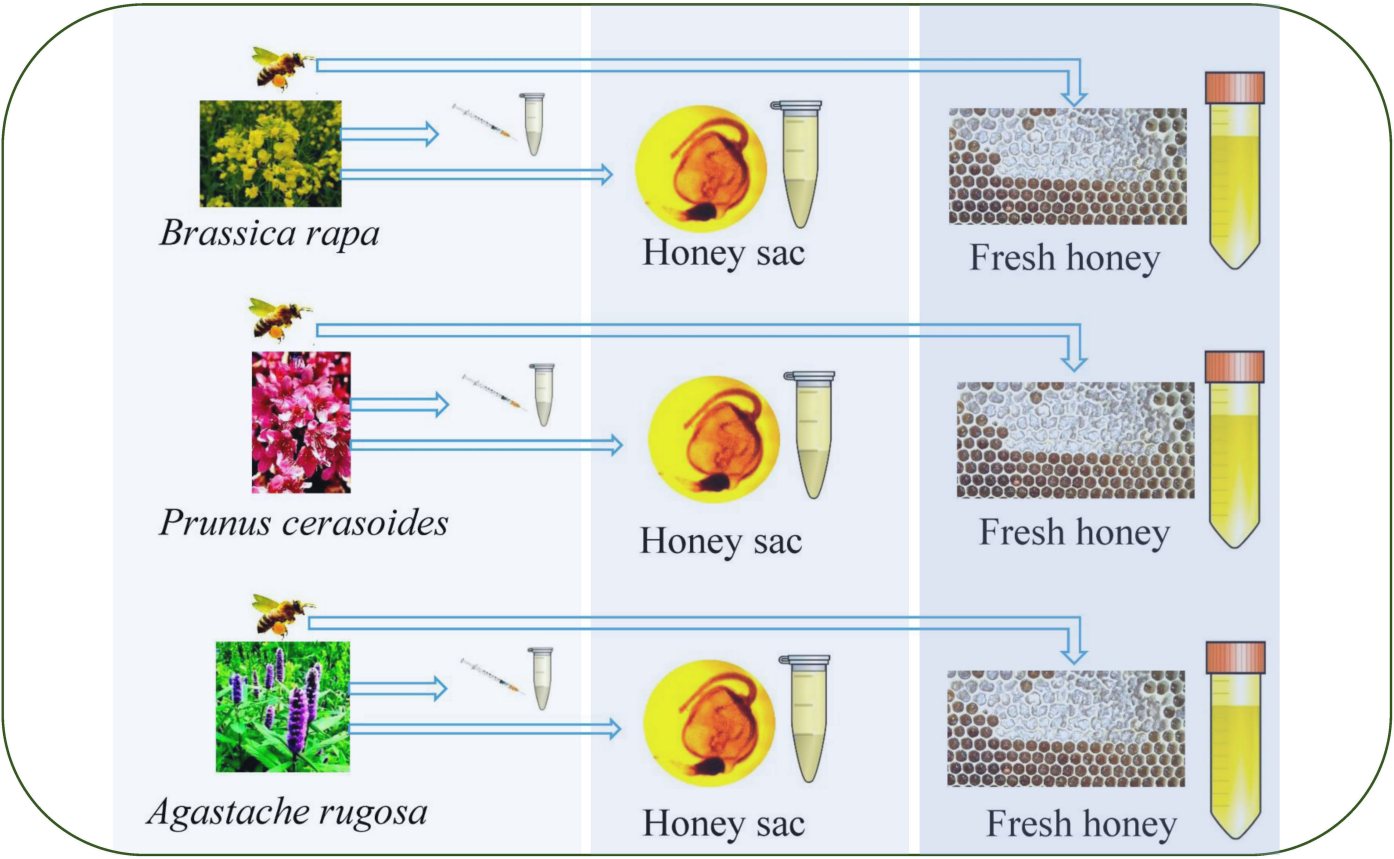
Sampling procedure from nectar to honey sac to fresh honey from the honeycomb. On the left side of the figure is the collection of nectar from different floral sources during the flowering period, in the middle of the figure is the collection of sacs from two honey bee species from three floral source areas, and on the right side of the figure is the collection of honey from two honey bee species from three floral source areas.

Through investigation, it was confirmed that there were no other floral plants that were flowering at the same time. Nectar was collected using a sterile capillary in a field near each apiary. In total, 38,142 *A. rugosa* (*Ar*), 297 P*. cerasoides* (*Pc*), and 9,810 *B. rapa* (*Br*) flowers were artificially collected and 9 mL of each nectar was collected for subsequent analyses. All samples were frozen immediately after collection, stored at –20°C, and screened within 2 months (23) (**Figure 1**).

#### 2.1.2 Honey sac sample collection

We collected honeybees from November to February of the next year, during the full bloom period of *A. rugosa*, *P. cerasoides*, and *B. rapa*, from three apiaries with *A. cerana* and *A. mellifera* in Mengzi (N23°29′-N24°22′, E103°18′-E103°33′, Al: 1256-1279m), Kunming (N25°07′, E102°45′ Al: 1958m), and Luoping (N24°49′-N24°57′, E104°18′-E104°22′, Al; 1555-1580m), Yunnan Province, Southwest China.

The colonies were all maintained using standard beekeeping practices. For surface sterilization, honeybees were suspended in 75% ethanol and then incubated in sterile water for 1 min each. All honey sacs were collected by aseptic excision into an aseptic tube. Samples were placed in separate sterile tubes containing 10 mL each of sterile physiological saline (0.9% w/v NaCl, 0.1% w/v Tween 80 and 0.1% w/v peptone) (23).For the two different species, *A. cerana* and *A. mellifera*, we collected 30 honey sacs from each colony, 10 of which were filled with a sterile sampling liquid tube. In total, 180 honey sacs were collected (**Figure 1**).

#### 2.1.3 Fresh honey sample collection

To obtain fresh honey samples, an empty honeycomb was placed in each of the three colonies of *A. cerana* and the three colonies of *A. mellifera*. After 24 hours, fresh honey was collected from each honeycomb placed using a sterile tube. Specifically, 3 mL of honey was collected from each colony. This resulted in a total of 9 mL of fresh honey collected from each honeybee species, a total of 18 mL. The collected honey samples were immediately stored at -20°C to preserve their integrity for subsequent experiments (**Figure 1**).

The samples were named according to the following abbreviations: LP: Luoping Yunnan province; MZ: Mengzi Yunnan province; KM: Kunming Yunnan province; 30××:Isolation bacterial strain number. Samples from LP were collected during the flowering season of *Br*. Samples from MZ were collected during the flowering season of *Ar*. Samples from KM were collected during the flowering season of *Pc*. All are the same in the text.

### 2.2 Culture media and methods

Tryptone soy broth (TSB) agar (24) and Man, Rogosa, and Sharpes (MRS) agar were used as culture media (23). Isolates were cultured aerobically in TSB medium at 37°C for 2–3 days or anaerobically in MRS medium at 37°C for 3–4 days using anaerobic flasks with Anaerocult (Merck, Darmstadt, Germany). From each growing surface plate containing 30–300 colonies each, 5–20 colonies of different morphologies were selected and each different colony was then subcultured to obtain pure isolates. A total colony count (CFU) was also performed when selecting the colonies.

### 2.3 DNA extraction

The collected bacteria grown on a plate were centrifuged at 10,000 rpm for 30 seconds at 4°C to obtain bacterial thalli according to the kit instructions. This process allowed us to collect 0.1–0.3 g of bacterial thalli. Pure DNA was then extracted from the bacterial thalli using the Tianamp Bacteria DNA Extraction Kit (Tiangen Biotech Co., Beijing, China) according to the manufacturer’s instructions.

### 2.4 PCR amplification of 16S rRNA genes and electrophoresis

PCR amplification of the 16S rRNA gene from each bacterial sample was performed using a thermal cycler (MJ Research, T100TM Thermal Cycler; Bio-Rad Co., Hercules, CA, USA). Each reaction mixture (final volume, 50μL) contained 4μL template DNA, 0.2μL each primer, 25μL 2× TransTaqTM II HiFi PCR SuperMix II (Transgen Co., Beijing, China), and 20.6 μL dH_2_O.

The universal oligonucleotide primers used to amplify the bacterial 16S rRNA gene were 27F (5′-AGAGTTTGATCCTGGCTC-3′) and 1387R (5′-GGGCGGTGTGTACAAGGC-3′). The PCR conditions included an initial denaturation of the DNA for 5 min at 94°C, then 30 cycles of denaturation of the DNA for 30 s at 94°C, annealing for 1 min at 58°C, and extension for 90 s at 72°C, followed by a final incubation for 7 min at 72°C (25, 26). PCR products were selected for sequencing.

### 2.5 Sequencing and phylogenetic analysis

#### 2.5.1 Sequencing

The purified PCR products obtained from the bacterial isolates were sequenced at Sangon Biotech Co. (Shanghai China) using 27F and 1387R primers. To determine the closest known relatives of the partial 16S rRNA gene sequences that we obtained, the sequences were queried against GenBank (National Centre for Biotechnology Information, Rockville Pike, Bethesda, MD, USA) using the Basic Local Alignment Search Tool (BLAST; http://www.ncbi.nlm.nih.gov/).

#### 2.5.2 Phylogenetic analysis

We compiled the 16S rRNA sequences from the isolated samples using SeqMan software. Next, the 16S rRNA and test strain sequences were edited using the BioEdit program and aligned using Clustal-W. After the deletion of regions containing ambiguous nucleotides, the distance matrix was calculated using BioEdit. Phylogenetic trees were constructed using the neighbor-joining method. To determine the stability of the phylogenetic tree, the sequence data were sampled 1,000 times for bootstrap analysis using MEGA version 11 with Kimura 2-parameter distances.

### 2.6 Methods for bacteriostatic testing

**Figure 2:**
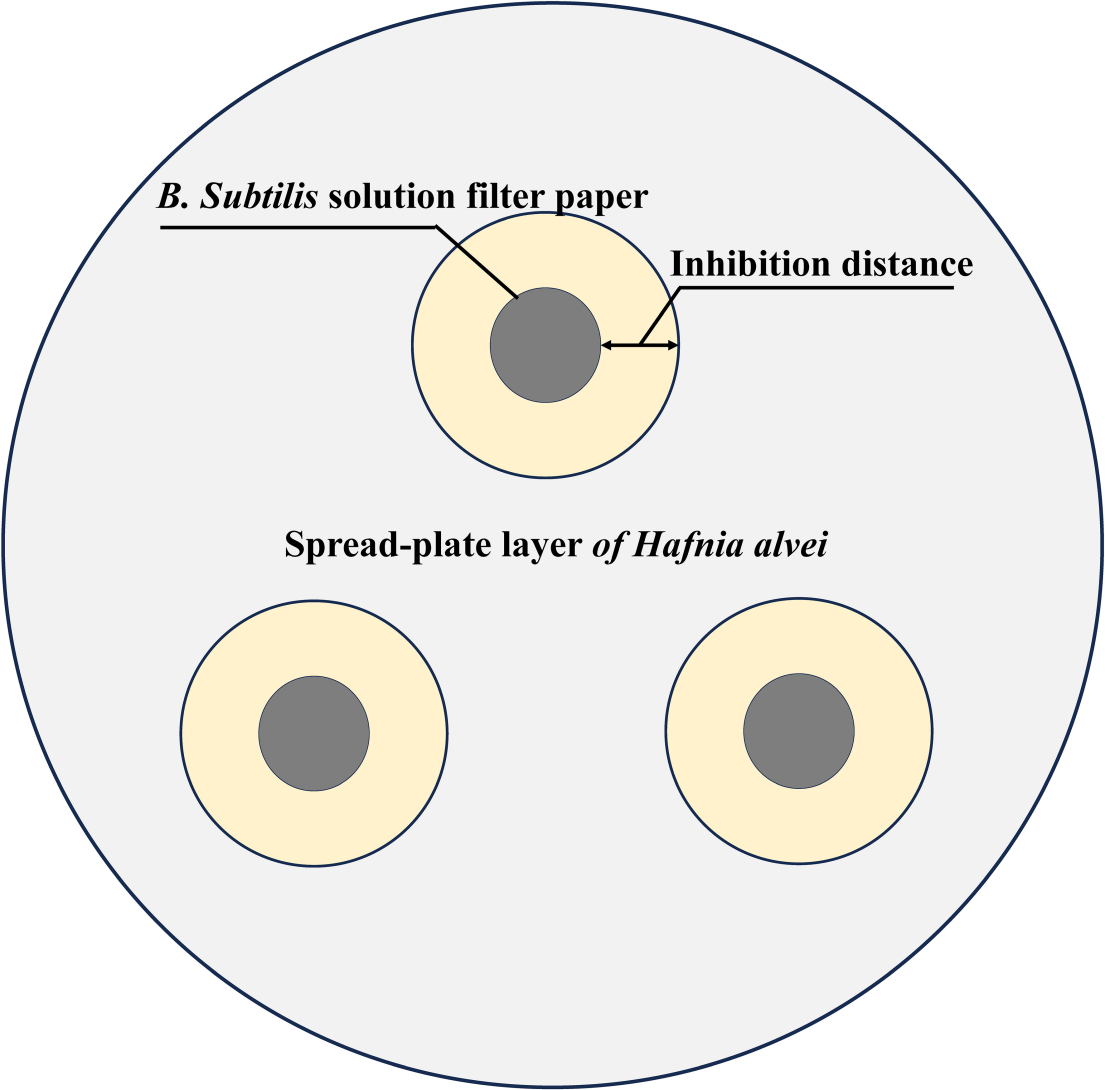
Schematic diagram of *B*. *subtilis* inhibiting *Hafnia alvei*. The gray area is the spread-plate layer of *H*. *alvei*, the black area is the *B*. *subtilis* filter sheet, and the yellow area is the bacteriostatic distance.

We selected three representative dominant strains from three floral source periods as test strains. The test strains were cultured with 0.1 g bacteria and 0.9 g sterile water for gradient dilution 10^-1^~10^-6^. Similar to the test strains, the pathogens were able to grow normally on TSB medium. Then, 0.1g of bacteria were collected by centrifugation, diluted with 0.9g of sterile water and 0.1mL were plated on TSB agar medium. The control plate was used as a standard for growth. Simultaneously, the treated test strains were placed on three pieces of 5 mm sterile filter paper equidistant from each plate as shown in **Figure 2**. The antibacterial distance was measured after 5 days of aerobic cultivation at 37°C (27).

### 2.7 Statistics

The number of bacteria was determined via two-way ANOVA, with floral sources (*A. rugosa*, *P. cerasoides*, and *B. rapa*) and honey sources (nectar, honey sac honey, and fresh honey) as fixed effects, while honeybee species was set as the random effect. Tukey’s HSD test was used as a *post hoc* test. All statistic were performed in R Studio (2022.02.3). The results of the antibacterial experiment were determined to be statistically significant using one-way ANOVA.

## 3 Results

### 3.1 Abundant bacteria were detected in nectar, honey sacs, and honey

**Figure 3:**
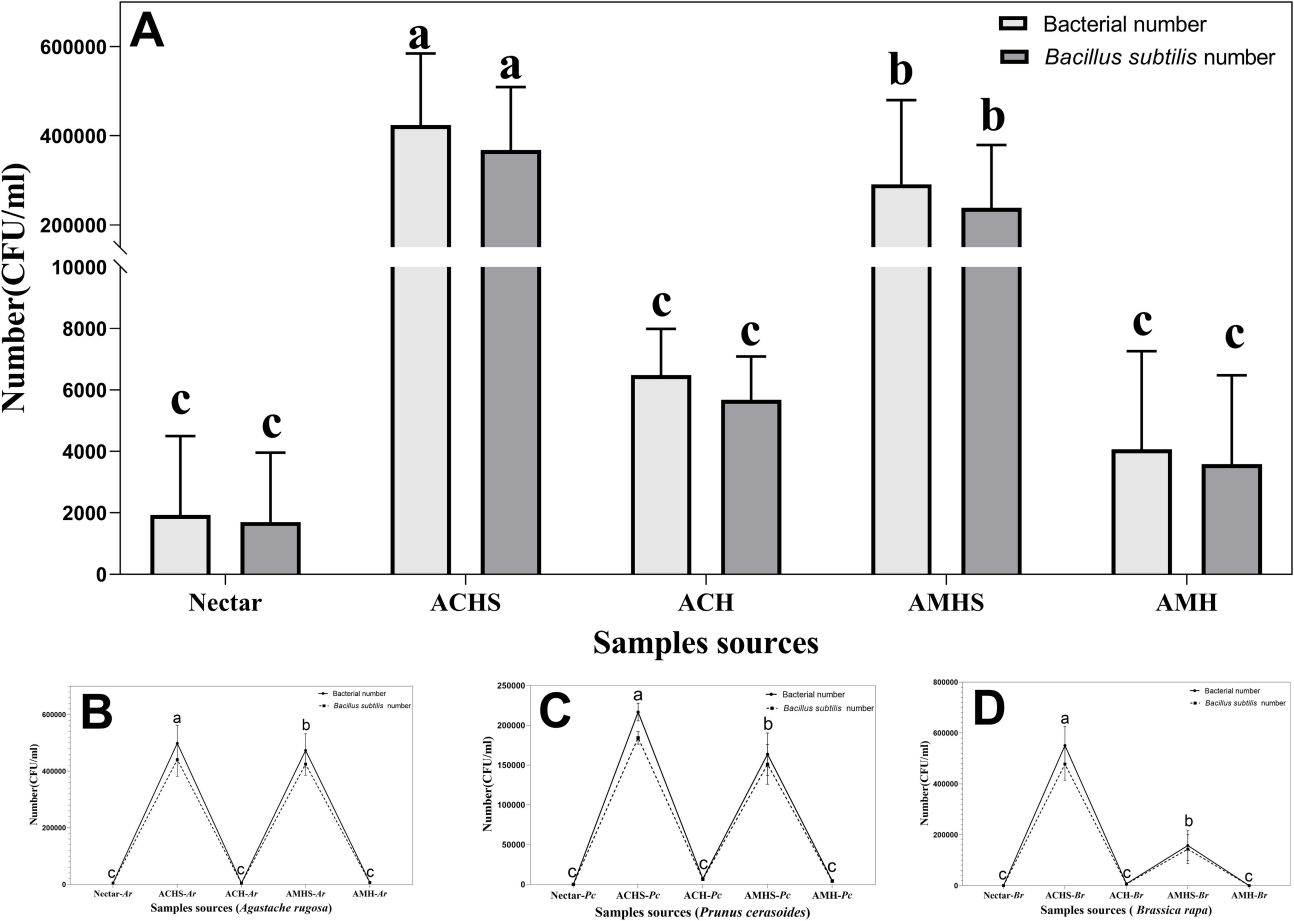
The total number of bacteria and *B*. *subtilis* between different sample sources and their interactions. **(A)** Plot of the number of bacteria in all samples during the winter flowering period of the floral source. Plot of the number of bacteria during the flowering period of *Agastache rugosa* **(B)**, *Prunus cerasoides* **(C)**, and *Brassica rapa* **(D)**. Different letters showed significant differences, *P*<0.05. ACHS, honey sac of *A*. *cerana*; AMHS, honey sac of *A. mellifera*; ACH, honey of *A*. *cerana*; AMH, honey of *A. mellifera*. Same below.

A sum analysis was performed on all samples and there was more bacteria in the honey sac than in nectar or fresh honey for both bee species (F_4,130_ = 100.918, *P*<0.001). The number of *B. subtilis* was also higher in the honey sac than in nectar and fresh honey (F_4,130_ = 99.258, *P*<0.001). *Post-hoc* comparison results showed that bacterial quantity was highest in honey sacs of *A. cerana* which was higher than in honey sacs of *A. mellifera* (Tukey’s HSD, *P*<0.001) (**Figure 3A**).

In the three different winter flowering stages, the number of bacteria in all samples changed in the same way. *B. subtilis* was the dominant bacteria in the samples and the number and total number of bacteria also changed in the same way. Similar trends of bacterial abundance were found in winter floral sources of *Pc* (F_4,40_ = 574.780, *P*<0.001), *Ar* (F_4,40_ = 416.315, *P*<0.001), and *Br* (F_4,40_ = 278.476, *P*<0.001), while the highest abundance was found in the honey sac samples of *A. cerana* compared with the honey sac samples of *A. mellifera*, followed by the nectar and honey samples (**Figures 3B–D**).

**Figure 4:**
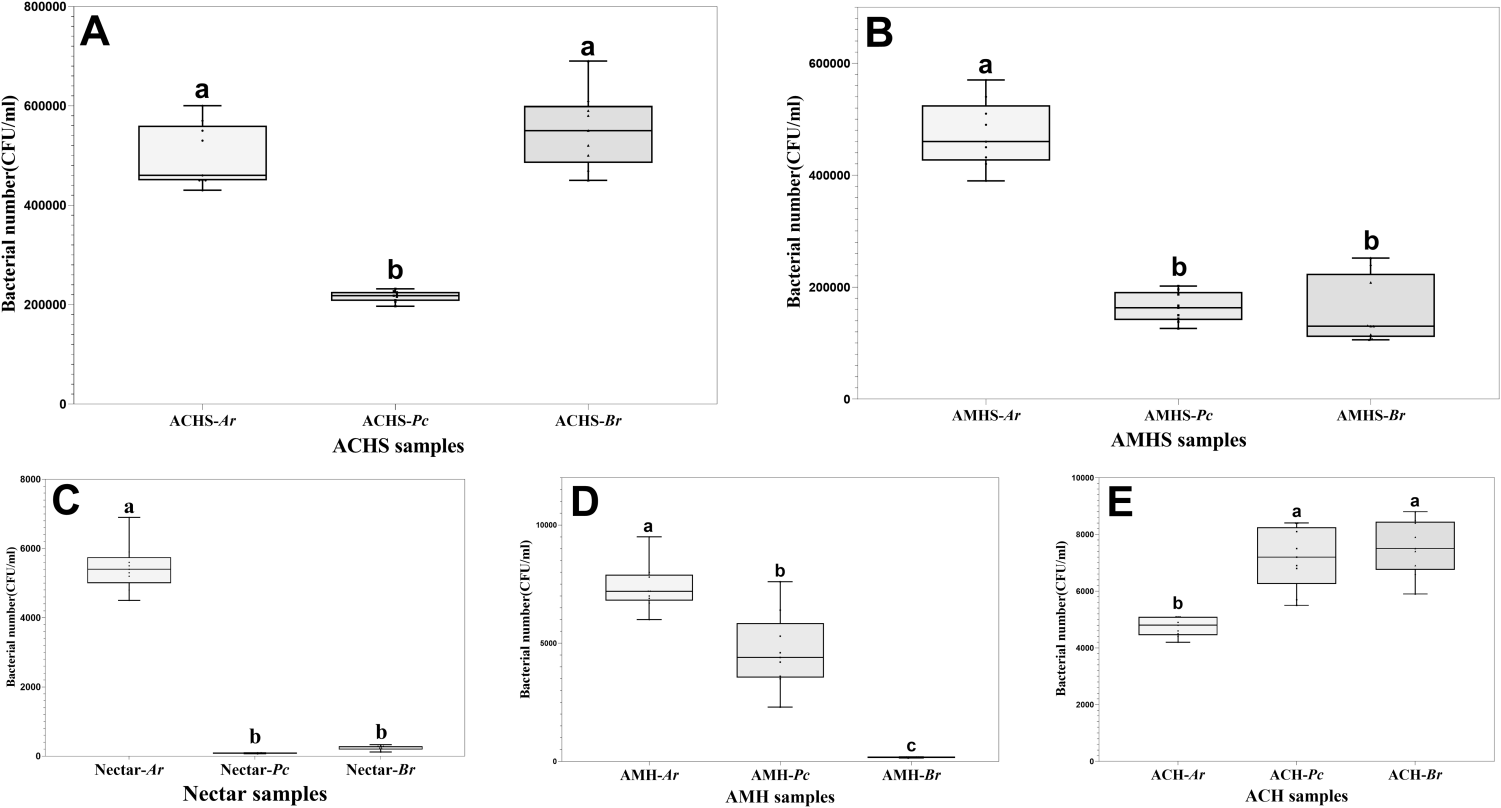
Comparison of bacterial number in honey sacs **(A, B)**, nectar **(C)**, and fresh honey **(D, E)** from bee colonies during winter flowering of *Ar*, *Pc*, and *Br*, respectively. Different letters show significant differences, *P*<0.05.

The number of bacteria was significantly different in the three winter flowering sources. The number of bacteria in the honey sacs of *A. cerana* at *Pr* anthesis was significantly lower than at *Ar* and *Br* anthesis (F_2,24_ = 89.750, *P* < 0.001). There was no significant difference between *Ar* and *Br* anthesis (*P*=0.097) (**Figure 4A**). The number of bacteria in the honey sacs of *A. mellifera* at *Ar* anthesis was significantly higher than at *Pc* and *Br* anthesis (F_2,24_ = 115.847, *P* < 0.001). There was no significant difference between *Ar* and *B*r anthesis (*P*=0.803) (**Figure 4B**).

The highest bacterial quantity of nectar was found in *Ar* compared with the other two winter floral sources (*P*<0.01), while no statistical difference was found between *Br* and *Pc* (*P*=0.400) (**Figure 4C**).

The number of bacteria in the honey collected by *A. mellifera* and *A. cerana* was the lowest and the difference between the other groups was statistically significant, except that the difference in the honey quantity of *A. cerana* at the flowering stage of *Pc* and *Br* was not significant (Tukey’s HSD, *P*<0.05) (**Figures 4D,E**).

### 3.2 Result of molecular bio-identification of the microbial strains

Bacterial isolation and identification in the nectar, honey sacs, and honey from the LP (*Br*), KM (*Pc*), and MZ (*Ar*) flowering seasons yielded 66 strains of different bacterial species. The strains were submitted to NCBI, and after comparison, information on similar strains and strain accession numbers from NCBI, and similarity ranges and degrees of similarity were obtained (**Table 1**). Other similar strains are also listed in **Table 1**.

**Table 1:** The most closely related strain types from the honey sacs, nectar, and honey.

Isolation and accession numbers from NCBI ^A^	Similar bacterial strain login number from NCBI ^B^	Similar interval ^C^	Sequence lengths and similarity ^D^	Other similar isolates and accession numbers from NCBI ^E^	Number and origin of identified strains Origin (number) ^F^
H-AMLP3107 (PQ158548)	*Acetobacteraceae bacterium* H14_5_2SCO_2_ 16S ribosomal RNA gene, partial sequence (KF599461.1)	7–1,244	1,234/1,238(99%)	H-AMMZ3059 (PQ158549), ACSKM3037 (PQ158547)	LP (1),MZ (2), KM (1)
ACSKM3033 (PQ160366)	*Acinetobacter lwoffii* strain P41 16S ribosomal RNA gene, partial sequence (MT323124.1)	19–1,187	1,169/1,169(100%)		KM (1)
AMSLP3070 (PQ160369)	*Agrobacterium tumefaciens* strain NPRH3 16S ribosomal RNA gene, partial sequence (MT383661.1)	44–1,243	1,200/1,200(100%)		LP (1)
N-KM3014 (PQ160368)	*Agrobacterium larrymoorei* strain MRC1261 16S ribosomal RNA gene, partial sequence (PP301774.1)	33–1,253	1,221/1,221(100%)	AMSKM3032 (PQ160367)	KM (2)
ACSLP3077 (PQ160377)	*Bacillus aryabhattai* strain ZJJH-1 16S ribosomal RNA gene, partial sequence (MT605510.1)	2–1,317	1,316/1,316(100%)	AMSKM3023 (PQ160378) H-AMKM3008 (PQ160379)	LP(1), KM (2)
N-LP3082 (PQ160376)	*Bacillus amyloliquefaciens* strain 3820 16S ribosomal RNA gene, partial sequence (MT538668.1)	21–1,328	1,308/1,308(100%)	AMSMZ3053 (PQ160374), H-AMKM3009 (PQ160375), AMSKM3020 (PQ160372), ACSKM3034(PQ160370), AMSLP3075 (PQ160373), ACSLP3071(PQ160371)	LP (4), MZ (7), KM (5)
ACSKM3042 (PQ160381)	*Bacillus subtilis* strain Q3 KT250124 16S ribosomal RNA gene, partial sequence (PP542510.1)	9–1,301	1,293/1,293(100%)	ACSLP3079 (PQ160382), ACSMZ3050 (PQ160383), AMSKM3025 (PQ160384), AMSLP3101 (PQ160385), AMSMZ3054 (PQ160386), H-ACKM3004 (PQ160387), H-ACHLP3109 (PQ160388), H-ACMZ3043 (PQ160389), H-AMKM3110 (PQ160390), H-AMLP3067 (PQ160391), H-AMMZ3046 (PQ160392), N-KM3012 (PQ160393), N-LP3068 (PQ160394), N-MZ3056 (PQ160395)	LP (154), MZ (122), KM (136)
AMSMZ3049 (PQ160380)	*Bacillus stercoris* strain M-5 16S ribosomal RNA gene, partial sequence (PP972774.1)	60–1,297	1,238/1,238(100%)		MZ(1)
AMSMZ3051 (PQ160396)	*Bacillus tequilensis* strain TRM58520 16S ribosomal RNA gene, partial sequence (PP978608.1)	77–1,255	1,179/1,179(100%)		MZ(1)
AMSLP3104 (PQ160397)	*Brevundimonas vesicularis* strain TK026 16S ribosomal RNA gene, partial sequence (MK045801.1)	21–1,163	1,143/1,143(100%)		LP(1)
N-MZ3064 (PQ160398)	*Curtobacterium oceanosedimentum* strain P120 16S ribosomal RNA gene, partial sequence (MT487608.1)	69–1,216	1,148/1,148(100%)		MZ(2)
N-MZ3065 (PQ160399)	*Curtobacterium pusillum* strain P124 16S ribosomal RNA gene, partial sequence (MT487611.1)	94–1,248	1,155/1,155(100%)		MZ(2)
ACSKM3038 (PQ160404)	*Enterococcus faecalis* strain CLA-AA-H229 16S ribosomal RNA gene, partial sequence (PP977882.1)	53–1,353	1,300/1,301(99%)		KM(2)
ACSKM3006 (PQ160400)	*Enterobacter cloacae* strain Remi_3 16S ribosomal RNA gene, partial sequence (MT507083.1)	8–1,149	1,138/1,142(99%)	AMSKM3022 (PQ160401), H-MKM3036 (PQ160403), H-ACKM3003 (PQ160402)	KM(4)
AMSKM3007 (PQ160480)	*Moraxella osloensis* strain TS5 16S ribosomal RNA gene, partial sequence (PP693046.1)	1–1,455	1,448/1,455(99%)		KM(2)
AMSKM3030 (PQ160405)	*Gluconobacter frateurii* gene for 16S ribosomal RNA, partial sequence, strain SL13-7 (AB819118.1)	23–1,262	1,225/1,241(99%)		KM(1)
N-MZ3058 (PQ160406)	*Lactococcus lactis* strain RS1 16S ribosomal RNA gene, partial sequence (PP068842.1)	80–1,293	1,214/1,214(100%)		MZ(1)
N-MZ3060 (PQ160407)	*Leuconostoc mesenteroides* strain 4601 16S ribosomal RNA gene, partial sequence (MT545098.1)	84–1,264	1,181/1,181(100%)		MZ(1)
ACSKM3001 (PQ151644)	*Leclercia adecarboxylata* strain KBD-4 16S ribosomal RNA gene, partial sequence (ON329817.1)	19–1,296	1,278/1,278(100%)		KM(1)
N-MZ3057 (PQ160408)	*Leuconostoc pseudomesenteroides* strain 4464 16S ribosomal RNA gene, partial sequence (MT544990.1)	65–1,290	1,226/1,226(100%)		MZ(1)
ACSKM3039 (PQ160409)	*Luteibacter anthropi* strain JSPC12 16S ribosomal RNA gene, partial sequence (OM319731.1)	21–1,307	1,262/1,288(98%)		KM(1)
N-KM3013 (PQ160410)	*Microbacterium hominis* strain 1P10AE 16S ribosomal RNA gene, partial sequence (EU977655.1)	31–1,274	1,241/1,244(99%)		KM(1)
AMSKM3029 (PQ160411)	*Neokomagataea thailandica* strain isolate 59 16S ribosomal RNA gene, partial sequence (OP595660.1)	13–1,242	1,230/1,230(100%)		KM(1)
ACSKM3002 (PQ160414)	*Pantoea cypripedii* strain T8H6 16S ribosomal RNA gene, partial sequence (MH011942.1)	1–1,213	1,209/1,213(99%)	H-ACKM3019 (PQ160415), AMSKM3028 (PQ160416)	KM(4)
AMSKM3031 (PQ160412)	*Pantoea ananatis* strain DJC4-3 16S ribosomal RNA gene, partial sequence (PP792809.1)	59–1,315	1,249/1,257(99%)	N-KM3015 (PQ160413)	KM(2)
N-KM3010 (PQ160417)	*Pantoea vagans* strain -Y14 16S ribosomal RNA gene, partial sequence (JX077090.1)	6–1,294	1,285/1,289(99%)		KM(2)
N-KM3011 (PQ160418)	*Pseudomonas psychrotolerans* strain LJBJ9 16S ribosomal RNA gene, partial sequence (PP651578.1)	27–1,320	1,293/1,294(99%)		KM(2)
AMSKM3026 (PQ160419)	*Rhizobium giardinii* strain 201-1 16S ribosomal RNA gene, partial sequence (OR673297.1)	21–1,257	1,229/1,237(99%)		KM(1)
AMSKM3027 (PQ160420)	*Sphingobium yanoikuyae* strain CG42 16S ribosomal RNA gene, partial sequence (MK618632.1)	1–1,228	1,228/1,228(100%)	AMSLP3069 (PQ160421)	LP(1),KM(1)
ACSMZ3061 (PQ160422)	*Staphylococcus epidermidis* strain CLA-AA-H299 16S ribosomal RNA gene, partial sequence (PP977939.1)	118–1,258	1,140/1,141(99%)	AMSKM3024 (PQ160423)	MZ(1), KM(2)
H-ACMZ3044 (PQ160424)	*Staphylococcus saprophyticus* strain HTK3 16S ribosomal RNA gene, partial sequence (OM049280.1)	81–1,258	1,178/1,178(100%)		MZ(2)
N-MZ3062 (PQ160425)	*Staphylococcus warneri* strain DK131 16S ribosomal RNA gene, partial sequence (MT642942.1)	99–1,246	1,148/1,148(100%)		MZ(1)
ACSKM3016 (PQ160426)	*Xanthomonas translucens* strain Tal22 16S ribosomal RNA gene, partial sequence (MH000694.1)	106–1,168	1,032/1,066(97%)		KM(2)

(A) Represents the strain and its accession number in NCBI; (B) Species and genus information of similar strains compared in NCBI; (C) Similarity interval; (D) Similar length; (E) other strains similar to the representative strain. (F) Number and origin of identified strains.

In total, 33 bacteria were isolated and identified from the nectar, honey sac, and honey samples from three different flowering sources collected by two bee species. Apart from *B. subtilis*, which was isolated from all samples, seven bacterial species were isolated and identified from *Br*. *B. amyloliquefaciens* was the only bacterial species isolated from the nectar and honey sacs of both bee species. Another seven bacterial species were isolated from only one of the sample sources. Furthermore, 13 bacterial species were isolated and identified from *Ar*, and other bacterial species were only isolated from one of the sample sources. In total, 22 bacterial species were isolated and identified from *Pc* and only 4 bacterial species were found in the honey sacs of both *A. cerana* and *A. mellifera* species, such as *B. amyloliquefaciens* and *Acetobacteraceae* bacteria. Other bacterial species were isolated from only one of the sample sources (**Table 1**).

### 3.3 Phylogenetic analysis of nectar, honey sac, and honey bacteria from three different nectar sources

**Figure 5:**
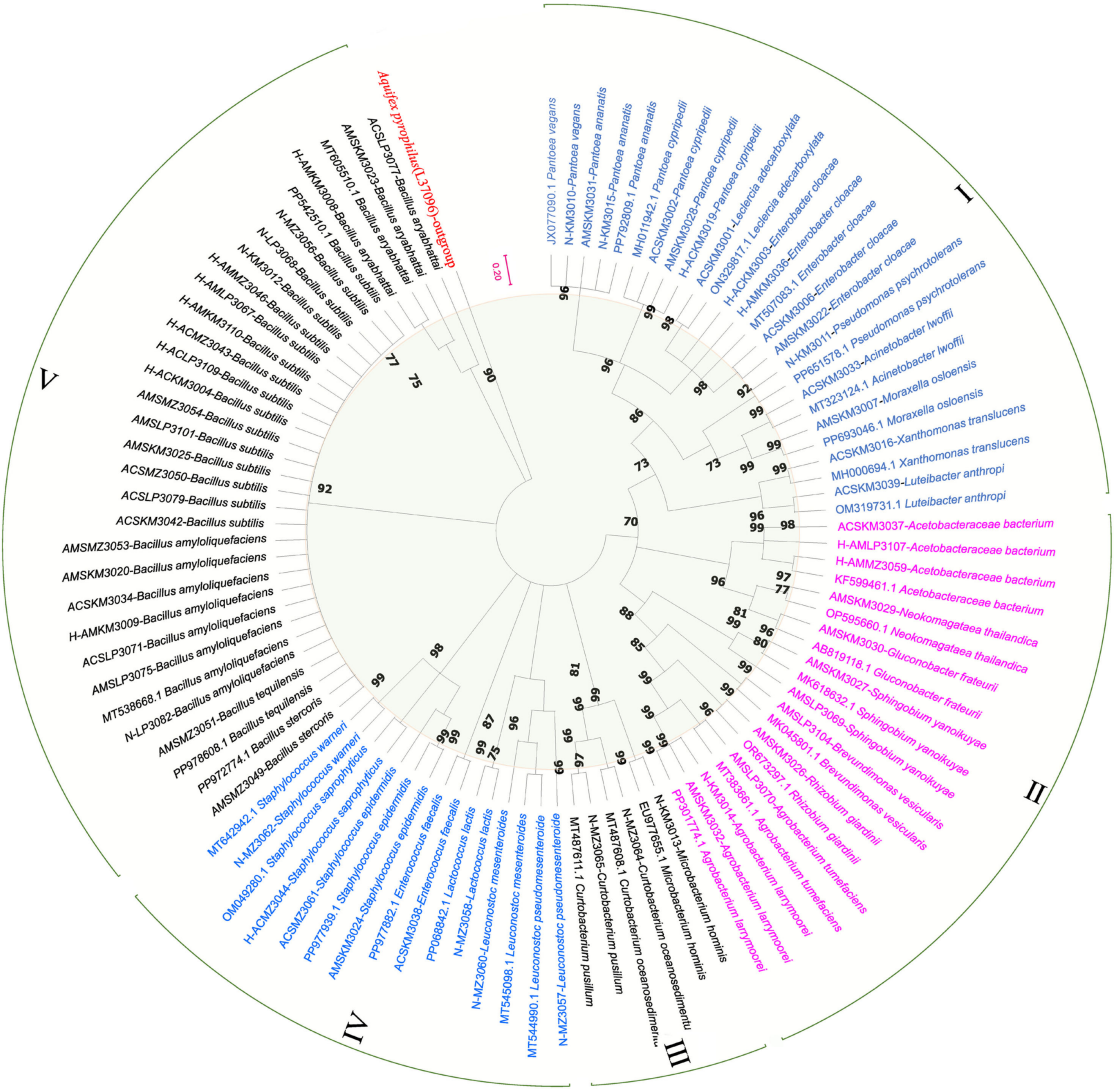
Phylogenetic tree of nectar, honey sac, and honey bacteria from three different nectar sources. The phylogenetic tree is based on a distance matrix analysis of 873 positions in the 16S rRNA gene and was constructed using ClustalW with the neighbor-joining method within the MEGA (11) package. Closely related types and reference strains are shown in parentheses together with accession numbers from GenBank. Bootstrap values based on 1,000 re-samplings display the significance of the interior nodes, and are shown at branch points. As shown by the bifurcation points, all five clusters have a bootstrap value of at least 70, and the scale of 0.2 represents a 20% evolutionary difference. Bacterial samples from different regions of the phylogenetic tree are grouped into five clusters (I-V), distinguished by color. The length of the branch is proportional to the genetic distance, and the longer the branch, the greater the difference between the samples.

Phylogenetic analysis clustered the samples from three different regions with different floral sources into five bacterial clusters (**Figure 5**). Cluster I: *Proteobacteria* and *Gammaproteobacteria*; Cluster II: *Proteobacteria*, *Alphaproteobacteria, Bacteroidetes*, and *Sphingobacteriia*; Cluster III: *Actinobacteria* and *Microbacteriaceae*; Cluster IV: *Firmicutes* and *Bacill*; Cluster V: *Firmicutes, Bacilli, Bacillales, Bacillaceae*, and *Bacillus*. The bacterium *Aquifex pyrophilus* was set as an outgroup (L37096).

This phylogenetic analysis reveals a clear division of the bacterial samples into five well-supported clusters, suggesting distinct evolutionary lineages or ecological adaptations within the sampled regions. The clustering pattern may reflect underlying environmental gradients, geographic separation, or host-specific associations that have shaped the diversification of these bacterial populations.

### 3.4 Bacterial cross-analysis of three types of nectar source samples in three regions

**Figure 6:**
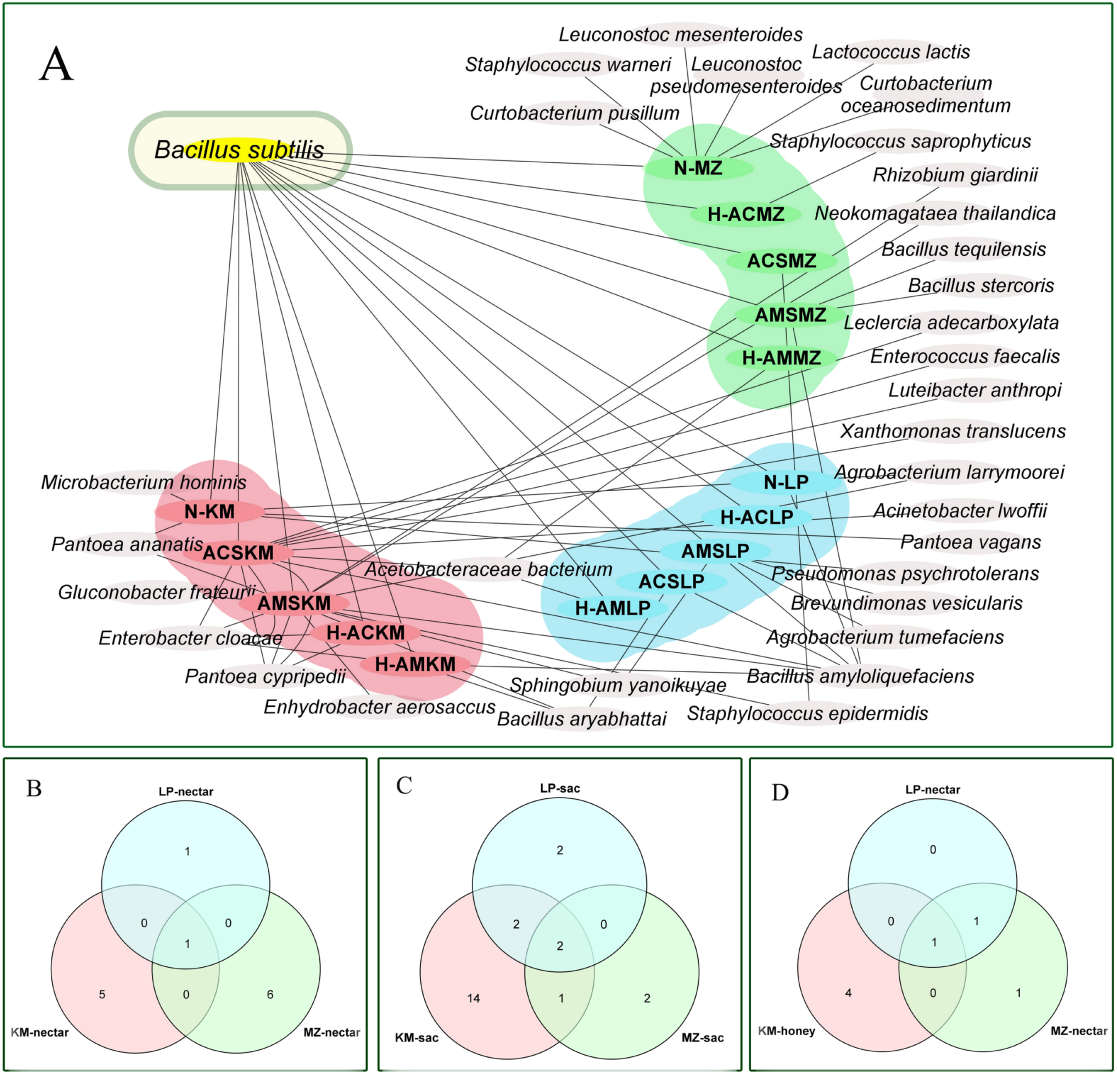
**(A)** Bacterial overlap map of three types of nectar source samples in three regions. **(B)** Venn diagram of bacteria in nectar from three regions. **(C)** Venn diagram of bacteria in honey sacs from three regions. **(D) **Venn diagram of bacteria in honey from three regions. (LP in blue, MZ in green, and KM in pink).

**Figure 6A** illustrates the patterns of bacterial distribution, highlighting both regionally specific and widely distributed types. The bacterial cross analysis showed that there were significant differences between the three different types of winter honey sources in the three different regions (LP, KM, and MZ). Only one common species of *B. subtilis* was present in all the samples, although in different regions. *B. amyloliquefaciens* was also found in some samples from LP and KM. Proteobacteria and Actinomycetes were found only in the KM region (*Pc*), but not in other regions.

**Figures 6B–D** show one type of nectar, two types of honey, and one type of honey that were common to the three regions, respectively.

### 3.5 Inhibition of *Hafnia alvei* by representative strains

**Figure 7:**
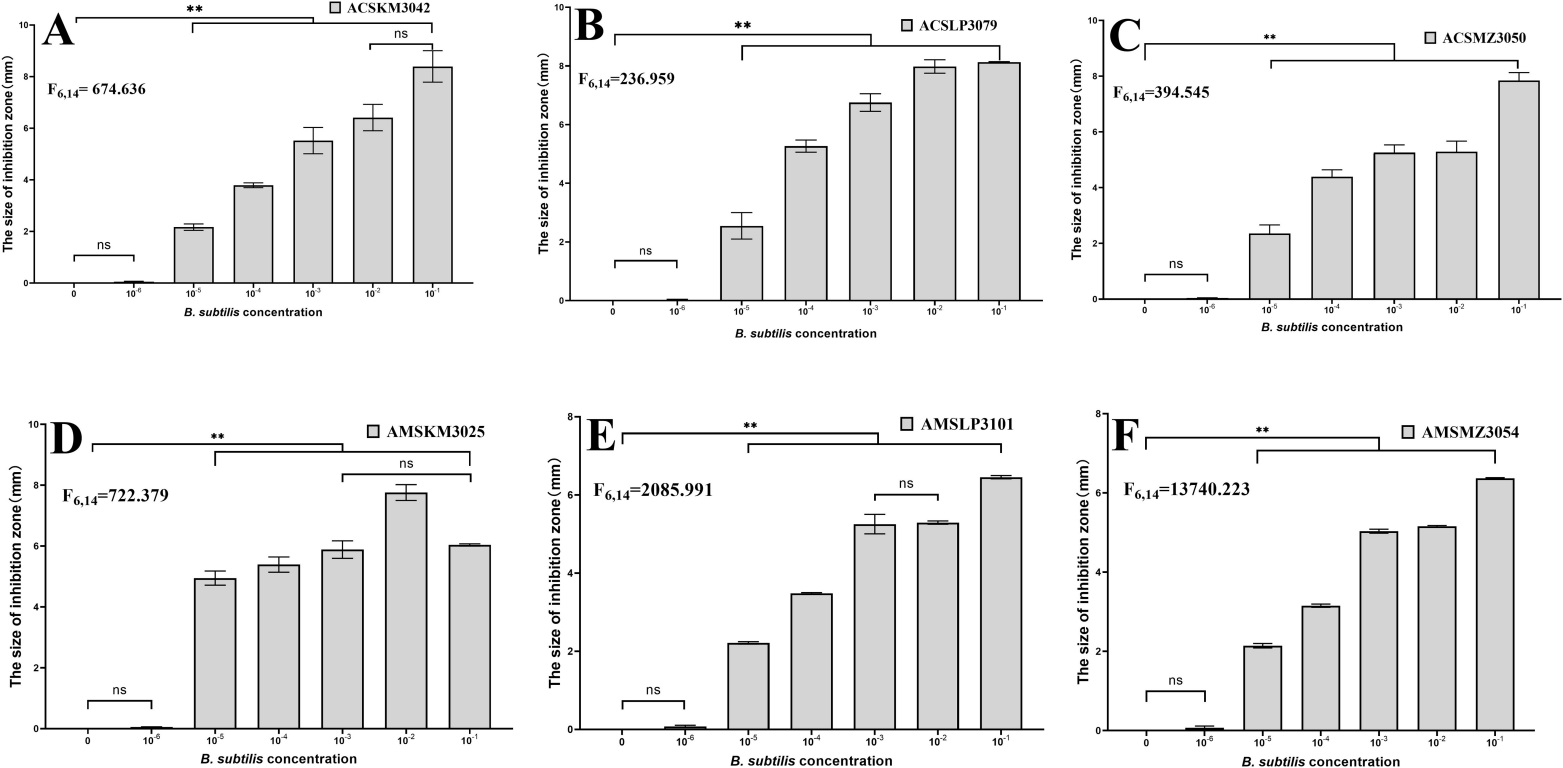
Inhibition of *Hafnia alvei* CMCC44102 by different dominant *B*. *subtilis* isolates from honey sacs. The six representative strains of *B*. *subtilis* from **(A–F)** are numbers ACSKM3042, ACSLP3079, ACSMZ3050, AMSKM3025, AMSLP3101, and AMSMZ3054 respectively. The horizontal axis is the H_2_O control and the concentration of *B*. *subtili*s, and the vertical axis is the inhibition distance. **, significant difference at p ≤ 0.01. ns, indicates no significant difference.

Six representative *B. subtilis* strains isolated from the honey sacs of *A. cerana* and *A. mellifera* during three winter flowering periods were tested in an inhibition of *Hafnia alvei* experiment, and the results showed that concentrations of 10^-1^–10^-5^ had a significant inhibitory effect on bacteria (*P*<0.001). The F values are in **Figures 7A–F**. In contrast to ACSKM3042 with concentrations of 10^-1^ and 10^-2^ (*P*=0.449) and AMSKM3025 with concentrations of 10^-1^ and 10^-3^(*P*=0.355), AMSLP3101, with concentrations of 10^-2^ and 10^-3^ (*P*=0.626), was not significant.

## 4 Discussion

The present study offers a detailed examination of bacterial diversity across the honey production process, from nectar collection to honey sac content and finally to fresh honey, during the winter flowering season of *Ar*, *Pc*, and *Br*. Our findings reveal a dynamic and complex microbial landscape that varies significantly with the bee species and the floral source, highlighting the intricate interplay between bees, their gut microbiota, and the environment.

The isolated and identified bacteria all have a similarity of >97%. It is currently widely accepted that when the homology of the 16S rDNA sequence is higher than 97%, it indicates a relationship within the same genus, and when it is higher than 99%, it indicates a relationship within the same species (28). Therefore, the identified strains have a high degree of reliability at the species and genus levels.

Only 33 bacteria were isolated and identified from the nectar, honey sac, and honey samples which was a relatively low identification rate compared to most other bee gut microbes or honey microbes studies (17). This could simply be a result of culture-based isolation constraints when compared with sequence-only-based identification (Olofsson et al., 2008; 17). This result is also consistent with the low number of bacteria found in honey sacs or mid-gut; while a large number of bacteria can be identified in the hind-gut (29). Due to the low temperature in winter, the growth and reproduction of bacteria would be inhibited, and the number and types of bacteria in the environment would be reduced (30). Therefore, due to the winter environment, nectar, honey sac, and honey bacterial phases will also change accordingly.

The most abundant bacterial quantities were identified in the honey sacs, suggesting that this stage of honey production is a hotspot for microbial activity. This abundance may be attributed to the bees’ regurgitative actions, which facilitate microbial fermentation and enzymatic processing of nectar (18, 23, 31). The differences in bacterial diversity between *A. cerana* and *A. mellifera* could be reflective of their distinct foraging behaviors and physiological adaptations to the winter environment (9). A previous study found that *A. cerana* starts foraging earlier and at lower temperatures than *A. mellifera*, even in the cold winter in Yunnan (32).

The floral source had a pronounced effect on the bacterial composition within the honey sacs, with *Ar*, *Pc*, and *Br* each contributing to a unique microbial signature. This variability underscores the importance of plant chemistry in shaping the bee gut microbiota and raises questions about how these differences may impact honey quality and bee health. *A. rugosa* is known for its antifungal, antibacterial, carminative, and antipyretic properties, and has been used as a traditional Chinese herbal medicine (33). It was no surprise that the number of bacteria was lower in *Br* than in the other two winter flowering flowers. The number of bacteria was higher in the honey sacs of *A. cerana* than *A. mellifera* after the bees foraged on *Br*, which differed from the bees that foraged on *Pc* or *Ar*, and could reflect that sympatric *A. cerana* are more adapted to these nectar sources with more secondary metabolites (34, 35).

The study’s focus on winter flowering plants provides critical insights into bee microbiota during a period of limited floral diversity. As previous studies have shown that the core bacteria are from the *Pseudomonas*, *Paenibacillus*, *Lonsdalea*, *Serratia*, and *Bacillus* genera, which are mainly analyzed in seasons other than the winter season (31). Liu et al. (9) analyzed the intestinal bacteria of *A. mellifera* in the winter season, and found its core bacteria were *Gilliamella*, *Bartonella*, *Snodgrassella*, *Lactobacillus*, *Frischella*, *Commensalibacter*, and *Bifidobacterium*, which are stable intestinal bacteria (9). *Agrobacterium* sp. is a bacterium that belongs to the *Rhizobiaceae* family and is normally associated with plants, not bees. It is found in the honey bee gut and may have been accidentally introduced into the honey sac by environmental factors, including bacteria from the genus *Agrobacterium*, or by bees during collection. This does not mean that these bacteria play an important role in the bee gut. However, in this study, the bees were sampled inside of the hive overwinter and the bees remained in the hive without any foraging activity. Our results differ from these previous studies either because of the different seasons, or different foraging activities.

The ability of bees to adapt to and process these alternative food sources is vital for their survival and the maintenance of colony health in the off-season. Understanding these adaptations is essential for developing strategies to support bee populations in the face of environmental challenges, such as habitat loss and climate change (36, 37).

The ubiquitous presence of *B. subtilis* across all sample sources is a noteworthy finding. As a known producer of antimicrobial substances and enzymes, *B. subtilis* may play a key role in the honey production process, potentially contributing to the stabilization and preservation of honey (18, 38). The similar high osmotic stress of the nectar, honey sac, and fresh honey samples meant that few bacteria could survive in it. *B. subtilis* existed in all the samples, which is consistent with a previous finding that osmotolerant bacteria can survive and even be transmitted from flower nectar to honey (39, 40).

A previous study showed that *B. subtilis* isolated from both honey sample and bee gut had high antimicrobial activity against the pathogens *Paenibacillus larvae* and *Ascosphaera apis* (19) and also showed antagonistic activity against the chalkbrood pathogen and pesticide degradation (41, 42).

The results showed that the representative *B. subtilis* in the samples had an inhibitory effect on *Hafnia alvei* at concentrations above 10^-5^, which could improve the resistance to disease of overwintering bees in the absence of honey sources and weak colony strength by the dominant *B. subtilis*, and suggested that *B. subtilis* enhances resistance against *H. alvei* infection, thereby contributing to bee health during the winter.

## 5 Conclusion

This study provides compelling evidence that the honey production process is significantly influenced by the bee species and the floral sources they exploit during winter months. The observed variations in bacterial diversity and abundance have implications for our understanding of bee health and the quality of honey produced. The findings underscore the need for a nuanced approach to beekeeping practices that consider the health of bees and the biodiversity of their gut microbiota. The significance of this work lies in its potential to inform apicultural practices and conservation strategies. By revealing the impact of winter floral resources on bee gut microbiota, the study offers a basis for selecting floral sources that support robust bee colonies. Additionally, the insights into the microbial dynamics during honey production can guide efforts to improve honey quality and the overall health of honeybee populations. As bees are critical pollinators for both agricultural and natural ecosystems, this research contributes to bee health and biodiversity conservation efforts.

## Data Availability

The datasets presented in this study can be found in online repositories. The names of the repository/repositories and accession number(s) can be found below: NCBI GenBank, accessions PQ158547–PQ158549, PQ151644, PQ160366–PQ160426, PQ160480.
